# The Dog–Owner Relationship: Refinement and Validation of the Italian C/DORS for Dog Owners and Correlation with the LAPS

**DOI:** 10.3390/ani11082166

**Published:** 2021-07-22

**Authors:** Giacomo Riggio, Patrizia Piotti, Silvana Diverio, Carmen Borrelli, Francesco Di Iacovo, Angelo Gazzano, Tiffani Josey Howell, Federica Pirrone, Chiara Mariti

**Affiliations:** 1 Department of Veterinary Sciences, University of Pisa, 56124 Pisa, Italy; c.borrelli2@studenti.unipi.it (C.B.); Francesco.diiacovo@unipi.it (F.D.I.); angelo.gazzano@unipi.it (A.G.); chiara.mariti@unipi.it (C.M.); 2 Department of Veterinary Medicine, University of Milan, 20133 Milan, Italy; patrizia.piotti1@unimi.it (P.P.); Federica.pirrone@unimi.it (F.P.); 3 Laboratory of Ethology and Animal Welfare (LEBA), Department of Veterinary Medicine, University of Perugia, 06126 Perugia, Italy; silvana.diverio@unipg.it; 4 Anthrozoology Research Group, School of Psychology and Public Health, La Trobe University, Bendigo, VIC 3552, Australia; T.Howell@latrobe.edu.au

**Keywords:** dog, owner, relationship, MDORS, C/DORS, LAPS, validation, Italian, bond, pet

## Abstract

**Simple Summary:**

The Cat/Dog–Owner Relationship Scale (C/DORS) is a questionnaire aimed to assess specific aspects of the pet–owner relationship. While the entire scale can be administered to both dog and cat owners, its validity and reliability have never been tested on dogs. Furthermore, validity and reliability of a scale may change depending on the respondents’ language and cultural background. Since the C/DORS was developed in English, we aimed to translate it into Italian and assess its validity and reliability on a sample of Italian dog owners. The response scale was modified to improve the variability of the owners’ responses. Overall, validity and reliability were good. The scale had the same three-factor structure (Perceived Emotional Closeness = PEC, Pet–Owner Interactions = POI, Perceived Costs = PC) reported for the original English version, although some items were removed because they did not fit the statistical model. The PEC subscale had the highest correlations with the subscales of the Lexington Attachment to Pets Scale. Finally, being a student owner was associated with higher PEC and POI scores. Conversely, owning a dog with behavioural problems was associated with lower PEC and higher PC. Owners whose dogs lived outdoors reported lower POI. Pet dog owners reported higher PEC than AAI dog owners.

**Abstract:**

The Cat/Dog–Owner Relationship Scale (C/DORS) can be administered to both dog and cat owners. However, the scale as a whole has never been validated on a sample of dog owners. Furthermore, it has never been translated into Italian. The aim of this study was to translate the C/DORS into Italian, modify its response scale in order to improve the degree of response variability, and test its validity and reliability on a sample of dog-owners. Exploratory factor analysis revealed the same three-factor structure (Perceived Emotional Closeness = PEC, Pet–Owner Interactions = POI, Perceived Costs = PC) as the original English version, although some items had to be removed because of low- or cross-loadings. The validity of the construct was confirmed by confirmatory factor analysis, by the correlations between each of the subscales and the C/DORS total score, and by the correlations with the Lexington Attachment to Pets Scale. Cronbach’s α values for each subscale were above acceptable levels. Student owners scored higher on PEC and POI than owners with other occupations. Owners of dogs with behavioural problems scored lower on PEC and higher on PC. Keeping the dog outdoor was associated with lower POI. Finally, pet dog owners scored higher on PEC than AAI dog owners.

## 1. Introduction

From the beginning of the 1980s, several scales have been developed to assess human perception of pet–owner relationship, such as the Pet Attitude Scale (PAS) [1], the CENSHARE Pet Attachment Survey [2], the Companion Animal Bonding Scale (CABS) [3] and the Lexington Attachment to Pets Scale (LAPS) [4]. The latter is probably the most widely used scale in human–animal relationship studies [5].

### 1.1. The Lexington Attachment to Pets Scale (LAPS)

The final version of the LAPS was developed by Johnson et al. in 1992 [4] with the aim to create a reliable psychometric tool to assess the level of owners’ emotional attachment to their pets. The LAPS items were developed using the Social Support Theory as theoretical framework. More specifically, the authors were interested in the affective aspect of the pet’s supportive role to the owner, which is considered the aspect of the relationship that most strongly affects human well-being and psychophysical health [6,7]. The scale is composed of 23 items with 0–3 Likert-scale response options that allow the respondents to indicate their level of agreement with each item. In their original study, Johnson et al. [4] found that the LAPS items related to three attachment dimensions that they named “General Attachment”, “People Substitution” and “Animal Rights/Animal Welfare”. Internal consistency was high for all dimensions, with Cronbach’s α being 0.90, 0.85 and 0.80 for general attachment, people substitution and animal rights, respectively. Even when considered as a whole, the scale had excellent psychometric properties (Cronbach’s α = 0.93) [4]. Similar factorial structure—although not identical in terms of item distribution within the three-factor model and reliability values—was obtained by Ramirez-Gonzalez et al. [8] in their validation study of the Spanish version of the scale. The LAPS has also been translated into German [9] and Italian [10]. In both languages, the scale showed good internal reliability, with high Cronbach’s α for the subscales of general attachment (German = 0.78, Italian = 0.93) and people substitution (German = 0.80, Italian = 0.82), but lower values for the animal rights/animal welfare subscale (German = 0.69, Italian = 0.65). Nonetheless, the LAPS has some conceptual limitations. Firstly, it focuses on the affective facet of the bond, not taking into consideration other aspects of the relationship that may be just as relevant. Secondly, having been developed for pet owners, in general, its items may fail to address some aspects of the pet–owner bond that may be unique to the relationship with specific pet species.

### 1.2. The Monash Dog–Owner Relationship Scale (MDORS)

More recently, in response to the need of having a scale that specifically assessed the dog–owner relationship, Dwyer et al. [11] developed the Monash Dog–Owner Relationship Scale (MDORS). Since its creation, the MDORS has been used in several studies [12,13,14,15,16,17]. Contrary to the majority of the previously used scales that mainly focused on the emotional facet of the pet–owner bond, the MDORS is a more heterogeneous tool that covers both affective and pragmatic aspects of the relationship. It is based on the Social Exchange Theory, which assumes that a relationship between two individuals is maintained or terminated depending on whether the benefits outweigh the costs or vice versa [18]. The MDORS items relate to three underlying dimensions of the dog–human relationship [11]. The Pet–Owner Interaction (POI) subscale reflects both general activities related to the physical care of the pet, as well as to more intimate activities, such as kissing, cuddling and hugging [11]. The Perceived Emotional Closeness (PEC) subscale is composed of items related to social support, affectional bonding, psychological attachment, companionship and unconditional love [11]. Finally, the Perceived Costs (PC) subscale includes items assessing negative aspects of the relationship or, in other words, the financial, social and emotional costs of caring for a pet [11]. The questionnaire has been translated into Swedish [19], Spanish [17], German [20], Danish [12] and Dutch [21]. In those cases where the translated versions were checked for differences from the original scale, some dissimilarities were observed. For instance, the principal component analysis performed by Schoberl et al. [20] on their German MDORS revealed a five-factor rather than the original three-factor model found by Dwyer et al. [11]. Handlin et al. [19] had to remove some items from their MDORS translation when they realised they were being misinterpreted by their sample of Swedish owners. Similarly, in the Dutch translation performed by Van Houtert et al. [21] some items did not fit the original model and consequently had to be removed. Moreover, when testing the subscales for internal reliability they found Cronbach’s α values that were well below acceptable levels (POI = 0.43, PEC = 0.19, PC = 0.19). As van Houtert et al. [21] suggest in their study, these findings highlight the importance of a thorough assessment of the validity and reliability of the MDORS when translated to new languages and targeting people from different cultures.

### 1.3. From the Monash Dog–Owner Relationship Scale to the Cat/Dog–Owner Relationship Scale (C/DORS)

While on the one hand the species-specificity of the MDORS may be considered as one of its strengths, on the other hand it represents a limitation for studies that aim to investigate the pet–owner relationship, in terms of balance between costs and benefits of pet-ownership, across different species [22]. Therefore, Howell et al. [22] developed the Cat/Dog–Owner Relationship Scale (C/DORS) by grouping into one single scale all the items from the MDORS and the additional items from the Cat–Owner Relationship Scale (CORS), a similar scale specifically designed for cat owners. The C/DORS is based on the same theoretical background and has the same structure as the MDORS. It consists of 32 items with a 1–5 multiple-choice response format. While the entire questionnaire can be administered to both dog and cat owners, only those items belonging to either the MDORS or the CORS must be included in the scoring process depending on whether the target species is a dog or a cat, respectively [22]. Therefore, to date, there is no unified scale to assess both dog– and cat–owner relationship in a cost–benefit balance perspective. Furthermore, the C/DORS as a whole has never been validated on a dog-owner sample. By including items from the CORS that are not present in the MDORS, the C/DORS may allow for a more detailed investigation of a wider range of dog–owner interactions. Finally, the C/DORS has not been translated into other languages, except for a study by Bowen et al. [23] on Spanish owners, in which some items were removed, and the response options were heavily modified to make the scale suitable to measure changes in the pet–owner relationship during the COVID-19 lockdown.

In light of all the critical points mentioned so far, the aim of this study was to validate an Italian version of the C/DORS among dog owners. We achieved this aim through a series of steps. Firstly, we translated a modified version of the C/DORS into Italian, to administer to a sample of Italian dog-owners. Secondly, we modified the existing C/DORS response options from a 1–5 scale to a 1–7 scale to increase response variability and, consequently, improve the psychometric properties of the scale. Thirdly, we assessed both the internal and the test–retest reliability of this modified version of the scale, as well as its validity through the confirmation of its underlying structure and its possible correlation with a widely used pet–owner relationship scale, namely the LAPS.

## 2. Materials and Methods

The study was approved by the Ethical Committee of the University of Pisa, Italy (protocol n 34/2020 in accordance with Directive 2010/63/EU).

### 2.1. Questionnaire Description

The questionnaire was composed of five different sections. Demographic data about the dog (breed, sex, age, neuter status, physical and behavioural problems, etc.), as well as the owner (age, gender, living environment, dog ownership experience, etc.) were collected in the first and second sections, respectively. The third section consisted of an updated version of the C/DORS [22]. The fourth section included all the 23 items from the LAPS [4]. Finally, in the fifth section, the respondents were asked, using free text, to provide three adjectives to describe their dog, as well as three adjectives to describe their relationship with their dog. An additional question was included to determine the respondents’ perception of the strength of their dog’s bond toward themselves. All questions were written in Italian.

### 2.2. Questionnaire Development

In order to provide an appropriate translation, both the C/DORS and the LAPS were first independently translated by two Italian native speakers who were proficient in English and had extensive knowledge of the dog–human relationship. Since the present study aimed to address dog owners only, the word “pet” used in the original versions of the C-DORS and the LAPS—which, on the contrary, refer to both dogs and cats—was replaced with the word “dog”. Afterwards, translations were compared, and incongruities were discussed to reach mutual agreement. As for the C/DORS, when the accuracy of the translation was in doubt, the opinion of the authors who originally developed the questionnaire [22] was requested. This latter step was not necessary for the LAPS, as no doubts on the meaning of the items arose during the translation process. Both questionnaires were then back-translated into English to reveal potential inconsistencies with the original versions. However, none were found, so the items were not further modified. The original English versions of the C/DORS and the LAPS along with their final Italian translations are reported in Tables S1 and S2, respectively.

As previously mentioned, in this study, an updated version of the C/DORS was used, meaning that the original 1–5 response scale was replaced by a 1–7 scale. This decision was taken considering the moderate to high skewness of some items reported by the authors of the original version. Furthermore, for all items, the response scale was inverted so that a higher score indicated a more positive relationship for all but 9 items, which were subsequently reverse scaled for the purpose of analysis. New C/DORS response options were discussed and agreed by all authors and are summarised in Table S3. All questions in the first, second and fifth section of the questionnaire were developed directly in Italian.

### 2.3. Questionnaire Distribution

An online version of the questionnaire was developed using Jotform^®^. An electronic link to the questionnaire was then shared on Facebook^®^, Menlo Park, CA, USA (http//www.facebook.com, accessed on 1 December 2020). Responses were collected over a one-month period, between December 2020 and January 2021. In order to complete the questionnaire, respondents had to (1) be dog owners, (2) be at least 18 years old and (3) have agreed to the informed consent. If they owned more than one dog, they were asked to consider the one they had been living with for the longest time.

### 2.4. Test–Retest Reliability

In order to assess test–retest reliability, 55 dog owners were asked to complete the questionnaire a second time, 15 days after the first completion. For both the first (T0) and the second (T1) time of participation, the selected participants were asked to write down a personal code—which had to be the same in both questionnaires—and report whether it was the first or the second time they were filling it out. The code allowed researchers to match questionnaires completed by the same respondents without providing any information on their identity.

### 2.5. Statistical Analysis

Analyses were carried out using R statistical software [24]. The packages psych [25], GPArotation [26], and lavaan [27] were used for factor analyses and the packages ordinal [28], rcompanion [29], and emmeans [30] for regressions.

Some of the levels of certain explanatory variables were aggregated for statistical analysis. Specifically, the dog’s breed was maintained if it was a breed reported at least five times; all other breeds were renamed as “other” in order to reduce the complexity of the model. For the same reason, we also summarised the household composition in three groups (single person, couple, three people or more), since we hypothesised that the number of people within the family may reflect in a different frequency of dyadic interactions. Similarly, the presence of other pets was summarised according to five categories: no other pets, other dogs only, cats only, both cats and dogs, and other type of pets. The source of the pet was classified based on factors predicting the development of behaviour problems and poor socialisation, such as a pet shops [31], those where the early experiences are generally unknown, such as shelters or pets found in the streets, and three categories typically associated with known and controlled early experiences, i.e., given by private individuals (friends, non-professional breeders), regulated breeders, or born at home; finally, dogs received as presents were allocated to a separate category.

Some variables where owners could choose more than one option were categorised into groups. Since we were interested in the way the owners’ lifestyle might be affecting their relationship with their dog, we categorised both the owner’s employment status, as well as the way they spent their time with the dog based on their likelihood to affect human–dog interactions. Jobs were organised based on whether the owners worked with their dogs or did not work with their dogs/were unemployed; in addition, responders could also be pensioners or students. For the activities with the dogs, the responses were categorised into working (e.g., hunting, search and rescue, shepherd), competitive dog sports (e.g., agility, flyball, obedience), amateur (they do training but do not compete), or no specific activities. Finally, the two questions regarding the presence of health conditions and behaviour problems were categorised into binary data (yes/no).

Data from the C/DORS were assessed in preparation for the explanatory factor analysis (EFA) used to examine the factor structure of the scale. After checking for low standard deviation (SD < 0.5), high skewness and kurtosis (above 6), the question “How often do you feel that having a dog is more trouble than it’s worth?” was removed from the dataset. Thus, 31 items were entered into this EFA. The results of parallel analysis (a statistical method used to determine the number of factors to retain in an exploratory factor analysis) and the exploration of the scree plot suggested three factors should be extracted. Therefore, on this basis, three factors were extracted with oblimin rotation and factor loading cut-off below 0.3. The obliquus rotation was chosen because we hypothesised a degree of overlapping between factors. Four items (“My dog gives me a reason to get up in the morning”, “How often do you feel that looking after your dog is a chore?”, “How traumatic do you think it will be for you when your dog dies?”, “How often do you take your dog to visit people?”) were excluded from the analysis because their loading was below the threshold, while one item (“How often do you take your dog in the car?”) was excluded due to cross-loading. Therefore, the remaining 26 items were analysed with another EFA with oblimin rotation.

Subsequently, we explored item response distributions of the LAPS and C/DORS questionnaires performing a confirmatory factor analysis (CFA). Given the ordinal nature of the data, we used the diagonally weighted least squares (DWLS) robust estimator. Several fit indices were considered to evaluate models: comparative fit index (CFI), Tucker-Lewis Index (TLI), root-mean-square error of approximation (RMSEA), and weighted root mean square residual (WRMR). Cut-off values for adequate fit for CFI and TLI were >0.90, RMSEA < 0.06 (Hu and Bentler, 1999), and WRMR < 1.00 [32]. The descriptive statistics and normality tests of the scales can be found in the supplemental information (Table 1). The reliability of both construct scales was also evaluated by Cronbach’s alpha (estimate of reliability). Finally, Spearman rho was used to assess test–retest reliability for the LAPS and the C/DORS by looking at correlations between T0 and T1 for each subdomain of the two scales. In addition, we measured whether the C/DORS subscales correlated with the overall scale.

To better understand the two scales, we investigated the correlations between the LAPS and C/DORS domains. We then used ordinal regressions to investigate the relationship between C/DORS scores, demographics (i.e., age and gender, level of education, job, household composition, previous experience with dogs), and the dog’s characteristics (i.e., signalment, health and behaviour anamnesis, housing situation, and presence of other pets). Before calculating the regression models, we used inferential tests to reduce model complexity. For each domain, the Kruskal–Wallis test was used with categorical data, the Mann–Whitney U test was used with binomial data, and the Spearman rho regression was used with continuous data in order to identify a relationship between the given variable and the sub-domain.

Ordinal logistic regression (OLR) models were then developed to assess the association between the Shared activities, Emotional Closeness, and Perceived Costs scores (dependent variables, Y) and each of the potential predictors (independent variables, X) for which there were significant differences in the preliminary analyses. Model fitting was tested using a likelihood ratio test and measured using the pseudo R2 calculated with Nagelkerke method. The odds ratio (ExpB) for the predictors was calculated to evaluate the strength of such relationships. A two-sided *p* < 0.05 was considered statistically significant.

## 3. Results

### 3.1. Demographic Data

#### 3.1.1. Participants

Overall, 1099 dog owners engaged with the study link, although only 1026 of them completed the questionnaire without missing any responses in the C/DORS and LAPS sections. When this occurred, the questionnaire was excluded from further analysis. In addition, 31 dog owners completed the questionnaire twice, providing a matchable code for test–retest reliability analysis.

The final sample (Table 2) consisted mostly of female participants (86%). Responders were aged 18–76 years (Mdn = 40 years). Participants were mostly from larger metropolitan areas, such as cities and towns (81%), they were rather equally distributed between levels of education and mostly they did not work with animals (63%). Their previous level of experience with dogs varied. Since not all owners answered all the demographic questions, the total number of responses for each question reported in Table 2 may vary from the total sample.

#### 3.1.2. Dogs

The sampled dog population (for full descriptive demographic results, see Table S4) was equally distributed between male (47%) and female (53%) dogs (Mdn age = 7 years, range <0.5–20), with similar proportions between intact (41%) and neutered dogs (59%). Half of the population consisted of mixed breed dogs (54%), with various degrees of representation of purebred dogs. Most of the dogs were privately obtained (40%) or acquired from a shelter (38%). Most of them were not trained (68%) and a smaller proportion engaged in training at home (36%). The dogs mostly lived at home (89%) and had no behavioural (82%) or medical issues (75%). There was a rather similar distribution of dogs living alone or with other pets, including other dogs or cats.

### 3.2. Factor Analysis and Questionnaire Validation

To evaluate the factor solution of the C/DORS, several well-recognised criteria for the factorability of a correlation were used. Firstly, we checked the data for outliers, looking at standard deviation, skewness, and kurtosis, and for low variance, removing item 19. Then it was observed that 24 of the 31 items correlated at least 0.2 with at least one other item, suggesting reasonable factorability. The Kaiser–Meyer–Olkin measure of sampling adequacy was 0.88, above the commonly recommended value of 0.60, Bartlett’s test of sphericity was significant (χ^2^ (465) = 10,890.33, *p* < 0.001), and the determinant of the correlation matrix was 0.00002. Given these overall indicators, factor analysis was deemed to be suitable with the remaining 31 items. Parallel analysis (PA) suggested a three-factor solution. The three-factor solution, suggested by PA, which explained 19% of the variance, was preferred because of its theoretical support [22], the scree plot, and the insufficient number of primary loadings and difficulty of interpreting additional factors. An oblimin rotation provided the best-defined factor structure. All items in this analysis had primary loadings over 0.3. Items with lower loading (items 2, 14, 19, 25, and 27) and with cross-loading (item 29) were removed, and the analysis was repeated. The factor loading matrix for this final solution is presented in Table 3.

The factor labels proposed by Howell et al. [22] suited the extracted factors and were retained. Factor 1 of the C/DORS was composed of items reflecting the facet of POI, such as spending time together or engaging in social activities, such as obedience training and dog sports. A higher score in this factor indicated more shared activities between the dog and the owner. Factor 2 was composed of items reflecting the PEC associated with companion dog ownership and relating to social support, bonding, companionship, and unconditional love. A higher score in this factor indicated that the owner perceived stronger emotional closeness to their dog. Factor 3 was composed of items reflecting the PC of companion dog ownership, addressing the negative aspects, including monetary costs, increased responsibility, and restrictions placed on the owner because of the dog. All items in this factor were reversed, so that a higher score in this factor indicated that the owner felt less affected by the negative aspects of dog ownership.

CFAs confirmed the structure of LAPS (χ^2^ (df = 227) = 655.25, *p* < 0.001, CFI = 0.98, TLI = 0.98, RMSEA = 0.04) and C/DORS (χ^2^ (df = 296) = 807.590, *p* < 0.001, CFI = 0.96, TLI = 0.96, RMSEA = 0.04) scales. Furthermore, Cronbach’s alpha demonstrated the Italian version of the LAPS (General Attachment α = 0.74; People substitute α = 0.84; Animal Rights α = 0.72) and C/DORS (Pet–Owner Interactions α = 0.84; Perceived Emotional Closeness α = 0.85; Perceived Costs α = 0.71) scales to have good internal reliability for all domains. Test–retest reliability (*n* = 31) was also deemed to be strong for the C/DORS PC domain, and for the LAPS General Attachment and Animal Rights domains. Test–retest reliability was very strong for the C/DORS POI and PEC domains, and the LAPS People Substitute domain (Table 4).

Finally, the overall C/DORS score (Mdn = 147, range = 84–188) had a very strong positive correlation with the POI domain (r = 83, *p* < 0.001), a strong correlation with the PEC domain (r = 77, *p* < 0.001), and a moderate correlation with the PC domain (r = 49, *p* < 0.001).

### 3.3. Correlations between LAPS and C/DORS

Spearman correlations indicated some degree of overlapping (positive correlations) between the LAPS and C/DORS scales (Table 5). Specifically, the C/DORS’ POI showed a moderate correlation with all three LAPS subscales. The C/DORS’ PEC was strongly correlated with the LAPS’ People Substitute and General Attachment, and moderately correlated with Animal Rights. The C/DORS’ Perceived Costs correlated only weakly with the LAPS domains.

### 3.4. Regressions

The model calculated for the dependent variable C/DORS POI explained 9% of the variance for this domain (Model fit: Nagelkerke Rp2 = 0.09, *p* < 0.001). A main effect without interaction of the fixed factors “occupation” (working with dogs, not working with dogs or out of jobs, retired, student), and “Living space” (indoors, outdoors only, indoors and outdoors) was observed (AIC = 6461.60, χ^2^_7_ = 83.78, *p* < 0.001, Table 6). In relation to the owner’s occupation, working with animals (MdnWorkAnimals = 58) and being retired (MdnRetired = 53) significantly decreased the odds of scoring high on the POI variable compared to being a student (MdnStudent = 57). Conversely, living both indoors and outdoors (MdnIndoor/Outdoor = 56) or indoors only (MdnIndoor = 56) increased the dogs’ odds of having a higher POI score, compared to living outdoors only (MdnOutdoor = 50) by a factor of 3.408 and 4.126, respectively (Table 7).

The model calculated for the dependent variable C/DORS PEC explained 6% of the variance for this domain (Model fit: Nagelkerke Rp2 = 0.06, *p* < 0.001). A main effect without interaction of the fixed factors “occupation” (Working with animals; Not working with animals/unemployed; retired; student), “activities with the dog” (AAI, sport, work, training at home, none), and “presence of behaviour problems” (yes vs. no) was observed (AIC = 5563.40, χ^2^_9_ = 60.45, *p* < 0.001, Table 6). It was observed that students (MdnStudent = 44) were more likely to score higher on PEC than unemployed owners and those who do not work with animals (MdnNotWithAnimals = 43) or owners who work with animals (MdnWithAnimals = 40). Similarly, owners of dogs that were not involved in any specific activity (MdnNoActivities = 42) reported a significantly greater PEC than owners of dogs involved in AAIs and assistance dogs (MdnAAI = 33). Finally, the odds for participants whose dogs had no behaviour problems of scoring higher on PEC (MdnNoProblems = 42) were 1.534 times greater than the odds for participants whose dogs had behaviour problems (MdnProblems = 40) (Table 7).

The model calculated for dependent variable C/DORS PC explained 6% of the variance for this domain (Model fit: Rp2 = 0.06, *p* < 0.001). A main effect without interaction of the fixed factor “presence of behaviour problems” (yes vs. no) was observed (AIC = 5301.10, χ^2^_1_ = 16.09, *p* < 0.001, Table 6). Post hoc pairwise analysis indicated that the PC was significantly associated with behaviour. Specifically, the odds for participants whose dogs had no behaviour problems to score high on the PC variable (MdnNoProblems = 43) were 1.6 times greater than the odds for participants whose dog had problems (MdnProblems = 41) (Table 7).

## 4. Discussion

The C/DORS is a relatively new scale that combines all the items from the MDORS [11], a widely used scale that assesses the dog–owner relationship, and the CORS [22] a similar tool that instead focuses on the cat–owner relationship. While the MDORS has been translated into different languages [12,17,19,20,21], it has not been translated or validated in Italian. With regard to the C/DORS, the researchers who first developed it [22] suggest that all the items be administered to both dog and cat owners, in multispecies studies. However, the scoring of the scale for each species involves only those items specific to the target species [22]. So far, the C/DORS as a whole has never been validated on a dog-only sample, in English or any other language.

The C/DORS version proposed in this study is not a mere translation of the original English version. Instead, we implemented a few changes aimed at improving the validity of the scale, to pave the way for a more suitable use of the C/DORS in cross-species comparisons, that does not require different scoring scales.

The Italian C/DORS for dogs used in this study retained the same factor structure reported for the MDORS, which consists of three factors named Dog–Owner Interactions, Perceived Emotional Closeness and Perceived Costs [11]. Nonetheless, several items (i.e., 2, 13, 14, 19, 25, 27 and 29) did not fit the original model.

Item 19 “How often do you feel that having a dog is more trouble than it’s worth?” was removed before analysis because of its high skewness. In this case, the great majority of the owners marked the response options with the lowest frequencies. The low response variability for this item may suggest that owners, regardless of the levels of interactions and emotional closeness with their dogs, and regardless of the perceived levels of practical and affective costs of dog ownership, tend to perceive their relationship with their dogs as overall more beneficial than detrimental. Another possible explanation is that our sample of respondents was biased towards owners that are more willing to spend their time completing a questionnaire on their dogs. These owners may be also more likely to care more about their relationship with their dogs [22]. On the contrary, owners that are indifferent or perceive their dogs as a burden may not volunteer for such a study. This type of bias, called volunteer bias or self-selection bias [33,34], is an issue that cannot be avoided in surveys that are openly advertised on the internet, such as this one. Therefore, caution should be made when interpreting the results in terms of their generalisability to the entire dog owner population [34].

Items 2, 14, 25, 27 and 29 were removed because they scored below the cut-off point of 0.3 or, in the case of the latter, because the difference between the loadings in two factors of the PA was lower than 0.1.

Item 2 (My dog gives me a good reason to get up in the morning) appeared to be a problematic item in Handlin et al. [19] Swedish translation of the MDORS, as well. However, in their case, the item was eliminated because it was unclear to Swedish respondents whether it referred to a positive or a negative “reason”. Although we overcame this problem by specifically referring to “good” reasons—as originally intended by Dwyer et al. [11]—this item showed a low loading on both POI and PEC factors.

Item 14 (How often do you feel that looking after your dog is a chore?) had similar low loadings in all three factors, suggesting the absence of a significant or even a preferential association with the underlying constructs of any of them.

Item 25 (How traumatic do you think it will be when your dog dies?) loaded just below the cut-off point in the PEC factor. In a previous study by Van Houtern et al. [21] this item caused interpretative issues as it loaded on POI rather than the original PEC. On the contrary, in this study it loaded on the PEC factor, although not enough to suggest an association with the other items in the same dimension. It is possible that the changes made to the response scale in our version of the C/DORS may have altered the answers to this item. In the process of widening the response scale from 5 to 7 points, the original scale, which went from “very traumatic” to “very untraumatic”, was converted into “very traumatic” to “a great relief”. Such modification was implemented for the following reasons. Firstly, there is no Italian translation for the word “untraumatic”. Secondly, the word “untraumatic” reflects the absence of a certain emotional response to a given event. Therefore, it may not make sense to grade the absence of a feeling. Thirdly, the original response options were structured as a bipolar scale (from very traumatic to very untraumatic) although they investigated a unipolar concept (presence/absence of a trauma). Therefore, considering the use of a bipolar scale for item 1, which is structurally similar to item 25, we decided to include opposite attributes at the extremes of the scale. However, considering the results obtained from the PA and the fact that none of the respondents considered the death of their dog to be relieving at any level, a unipolar scale going from “not traumatic at all” to “extremely traumatic” may be a better choice in future studies. Furthermore, finding an appropriate antonym for words with complex meaning, such as “traumatic” may not be an easy task, and the word “relieving” may not have the opposite meaning of the word “traumatic” in absolute terms. In fact, in some instances, the loss of a dog may be both traumatic and relieving, such in the case of a severely or chronically ill animal.

Item 27 (How often do you take dogs to visit people?) did not seem to be a problematic item in previous translations of the MDORS. However, it should be taken into account that our questionnaire was distributed during the COVID-19 pandemic. Contrary to a previous study by Bowen et al. [35], which used the C/DORS to investigate the effect of confinement on the dog–owner relationship, we did not remove this item from the scale because a total lockdown was no longer in place during our data collection period. However, it seems plausible that both the ongoing partial social restrictions (e.g., curfew, gathering limitations) and the fear of contracting the virus may still have affected our respondents’ social interaction dynamics [36] and, as a consequence, their response to this question. Future research should investigate this possibility by replicating the survey after COVID-19 restrictions have fully lifted.

Item 29 (How often do you take your dog in the car?) was removed as it cross-loaded in the POI and the PEC factors. In the Swedish translation of the MDORS, Handlin et al. [19] removed this item in consideration of the fact that not all owners have a car and, as a consequence, their response could be biased. Furthermore, as for item 27, the response to this question may have been altered by actual or self-imposed travel and movement limitations due to the COVID-19 pandemic.

Item 13 (How often you tell your dog things you don’t tell anyone else?) was not removed but loaded primarily on a different factor than in the original MDORS, that is POI instead of PEC. The same issue was reported by Van Houtern et al. [21] following the translation of the MDORS from English to Dutch. As they suggested in their study, the term “often” implies frequency, which may connect the related item to the POI factor, which measures actual frequencies of interaction, rather than to the PEC factor, that relates to an abstract affective dimension.

As mentioned at the beginning of this paragraph, the C/DORS, as originally developed by Howell et al. [22], is the combination of two species-specific pet–owner relationship scales, namely the MDORS for dog owners, and the CORS for cat owners. While some items apply to both dogs and cats, others are specific for a single species. When cross-species comparisons are desired, the authors propose to administer the entire questionnaire to the owners of both dogs and cats, and then calculating the score on different items according to the target species. Although non-species-specific scales for the assessment of some aspects of the pet–owner relationship already exist in the anthrozoological science panorama—e.g., the LAPS [4], the PAS [1], the CABS [3]—none of them investigate the relationship from a cost–benefit perspective. Therefore, in order to take a first step towards the development of a common C/DORS for dog and cat owners that could be suitable for cross-species comparisons, we included, in the validation process performed in this study, all the cat-specific items from the original C/DORS, which we adapted for and administered to dog owners (i.e., items 9, 15, 21 and 26). When applied to dog owners, all of these items loaded on the same factor as for cat owners, that is POI [22]. While this may represent a first positive result for the development of a C/DORS scale that is equally valid for dog and cat owners, future studies should focus on addressing the role of those items that, although included in this dog-adapted version of the C/DORS, when administered to cat owners may not fit the same model (e.g., load on different factors, or not load on any factors above acceptable levels).

In order to assess the reliability of the C/DORS for dogs we assessed the internal consistency of each of the identified subscales. Compared to original MDORS, the Cronbach’s α values appeared to be higher for the POI subscale (C/DORS = 0.84, MDORS = 0.67), similar for the PEC subscale (C/DORS = 0.85, MDORS = 0.84) and lower for the PC subscale (C/DORS = 0.71, MDORS = 0.84). Nevertheless, contrary to the MDORS, Cronbach’s α values were above acceptable levels for all the subscales. As the CDORS investigates aspects of the relationship that one may assume do not change over a short period of time, we also tested the C/DORS for test–retest reliability, which was found to be strong for all the subscales.

The construct validity of this new scale was also supported by the confirmation of the original three-factor structure obtained through CFA, as well as by the significant correlation between the three subscales and the CDORS total score. For the latter, it should be kept in mind that PC scores are reversed and therefore a negative correlation with this subscale and the total score is what we actually observed.

In consideration of the LAPS’ widespread use in human–animal relationship studies, we assessed the possible correlations between its subscales and the C/DORS subscales, with the aim of providing additional evidence of the C/DORS validity. Before doing so, the LAPS original three-factor structure was tested and confirmed through CFA. Furthermore, the internal consistency of each dimension was verified. Although lower than those reported for the original English version, internal consistency was still found to be acceptable to good for all dimensions. Results from the correlations observed with the LAPS dimensions provides further confirmation on the validity of the C/DORS. In fact, the highest significant correlation was found between the C/DORS’ PEC subscale and the LAPS’ People Substitute (PS) subscale. This result may be explained by the fact that both dimensions conceptually focus on the owner’s perceived role of the pet as emotional support (e.g., LAPS: “I love my dog because he/she is more loyal to me than most of the people in my life”, CDORS: “If everyone else left me, my dog would still be there for me”). Overall, among the CDORS subscales, the PEC had the highest correlations with all three LAPS subscales. This is not surprising since the LAPS, as its full name suggests, was conceived to assess the strength of the pet–owner attachment, intended as the emotional bond that ties two individuals. In fact, as Johnson et al. (1992) report in their original paper, all of the LAPS items were selected for their emphasis on the respondent’s affection toward the pet. On the contrary, the CDORS is a much more heterogeneous scale, comprising items that may not necessarily relate to the affective facet of the relationship. This is especially true for the PC subscale that includes items such as “My dog makes too much mess”, “There are aspect of owning a dog I don’t like” and “My dog costs too much money”. This may also explain why, amongst the three C/DORS subscales, the PC had the lowest correlations with all the LAPS subscales.

Some demographic factors of both owners and dogs were found to significantly predict the owner’s responses to the C/DORS subscales.

As for the PEC, student owners reported significantly higher scores than both owners who work with animals and owners who do not work with animals, as well as owners who are unemployed. While we find it hard to find a clear and comprehensive explanation for this result, especially in relation to the lower scores obtained by owners who work with animals, we can discuss those variables we believe may have led to this finding. First of all, we should consider the possibility that our sample of student owners was biased towards students from Veterinary Sciences courses because of the social media channels we used to distribute the questionnaire. However, our questionnaire did not discriminate between students from different courses, hence our hypothesis cannot be supported by scientific data. Secondly, the respondents’ age may also have played a role in the PEC scores. In fact, Kellert et al. [37] found that people between 18 and 35 years of age have a significantly greater humanistic attitude towards animals than people aged between 36 and 55 years and even greater than people aged over 56 years. In other words, the attitude of young adults towards animals is characterised by greater emphasis on the value of the animal as an individual and on the affective facet of the human–animal relationship, especially in relation to pets [37,38]. Furthermore, previous studies suggest that emerging adults—a term used to identify people aged between 19 and 29 years—tend to regard their dogs as either children or family members [39]. However, the perceived level of the pet’s inclusion within the family unit appears to be flexible as it tends to decrease over the course of the owner’s life, along with the changes in personal priorities and the beginning of parenthood [40]. However, this explanation remains speculative as the regression analysis did not reveal a direct effect of owner’s age on PEC scores. Finally, we should take into account that social restrictions implemented by the Italian government during the period of data collection, may have had a particularly negative psychological and emotional impact on this category of owners, as suggested by recent studies on Spanish [41] and American university students in COVID-19 times [42]. As a consequence, students may have relied on their dogs as a source of emotional support even more intensely than they have been observed to do in normal environmental and social conditions [43].

We do not have either a scientific or a logical explanation for the finding that owners of dogs involved in animal assisted interventions (AAIs) obtained lower scores on PEC compared with owners of dogs that did not perform any activities. It is possible that this result was biased by the low number of dog–owner dyads engaging in AAIs (*n* = 9), in our sample.

The association between ownership of dogs with behavioural problems and lower scores on the PEC is more straightforward. Hoffman et al. [44] found that owners of dogs with separation anxiety or generally misbehaving dogs reported lower levels of attachment. As reported by Buller [45], owners of problematic pets may experience a wide range of negative and conflicting emotions such as sadness for seeing their pets suffering, frustration for not being able to help them and anger for the negative effect the dog’s behaviour has on their daily life. Such negative emotions are likely to affect the owner’s perception of the dog as a source of emotional support.

As for the POI subscale, owners who worked with animals and those who were retired reported a lower frequency of interaction compared to owners who were students. A similar interpretation to the one provided for the association between the “job” demographic variable and PEC scores, can also apply to this finding. In fact, several items belonging to the POI subscale are, to a certain degree, the expression of emotional closeness (e.g., “How often do you kiss your dog?”, “How often do you cuddle your dog?”, “How often do you hug your dog?”). Indeed, other practical factors, such as the actual amount of time the owner can dedicate to the dog in daily life, may affect the frequency of pet–owner interactions. In this regard, it must be said that the daily routine of animal professionals, especially veterinarians, has not been substantially affected by COVID-19 restrictions, as it is the case for other categories of worker. In fact, in Italy, medical treatment for animals in need was guaranteed throughout the pandemic. On the contrary, attendance to in-person classes was suspended at the time of the data collection. This may have given student owners a greater amount of time to interact with their dogs. As for retired owners, it is possible that the reduction in mobility and daily activities that can occur along with the aging process [46,47] may have played a role in the lower frequency of dog–owner interactions observed in this study. However, we did not have a pool of elderly owners since only the 8% of the respondents were in their 60s, only the 0.2% were in their 70s and none in their 80s or above. Thus, like for PEC scores, this finding may also be explained by the greater emphasis that younger owners put in the emotional aspects of the dog–owner relationship [37], which could in turn lead to a higher frequency of kissing, hugging, cuddling, petting and shared experiences. However, since we did not find a direct effect of age on either POI or PEC scores, nor we investigated owners’ health issues that may have compromised their ability to interact with their dogs, this explanation requires further investigation in the future.

Unsurprisingly, owners whose dogs lived indoors or had free access to both indoor and outdoor spaces reported a higher frequency of interactions compared to owners of dogs that had no access to indoor space. This finding is in line with those from Shore et al.’s study [48], in which owners of yard dogs reported lower levels of affiliative, playful and physical care-related interactions compared to owners of house dogs. However, the relationship between these two variables does not imply causation. While it may be obvious that owners whose dogs live outdoor have fewer opportunities to interact with them, it is also plausible that owners who already are less motivated to interact with their dogs are also more likely to deny them indoor access.

As for the PC subscale, scores were higher for those owners who reported their dogs to have behavioural problems, regardless of their nature. Accordingly, Van Herwijnen et al. [16] reported higher perceived costs in owners of aggressive and disobedient dogs. Furthermore, this result is in line with previous studies that report dog behavioural problems to be one of the most frequent reasons for relinquishment [12,49,50,51]. Living with pets with behavioural problems may be emotionally and practically challenging as it is likely to lead to increased costs for professional intervention, increased difficulties in managing the pet in both public and private environments and reduced opportunities for social life [45].

Limitations of this study mainly relate to its nature as a questionnaire-based research. As mentioned above and as reported by Howell et al. [22] in their study on the CORS, self-selected participants may bias the sample towards owners that care more about their pets and have an overall more positive perception of the relationship with them. This may also explain why our sample consists mainly of female owners as well as owners residing in urbanised areas. Greater female involvement in human–animal relationship studies is not uncommon as women have been found to be more empathetic than men towards animal-related issues [52,53,54,55]. Similarly, people living in urban areas are reported to have a more positive attitude towards animals than people coming from a rural background [56]. The great number of dogs that were reported to live indoors is likely to be a consequence of the greater proportion of respondents living in urban areas.

Another important consideration should be made on the timing of data collection, which occurred during the COVID-19 pandemic. Although previous studies that investigated the effects of the pandemic on dog–owner relationship focused on the lockdown period [35], the partial social restrictions ongoing in Italy during the time of our study may still have affected our results. A similar investigation carried out after people’s life will return to normality may confirm or confute this possibility.

## 5. Conclusions

In conclusion, the C/DORS for dogs used in this study appears to be a valid and reliable instrument to assess Italian owners’ perception of the relationship with their dogs. A major difference from previous similar questionnaires, such as the MDORS and the CORS, is the wider range of response options that may help increase the response variability to all items. Furthermore, this scale seems to overall improve the psychometric properties of the original MDORS, as witnessed by Cronbach’s α values above acceptable levels for all the subscales. Therefore, we recommend its use even in single-species investigations. Similar studies carried out on samples from different countries are necessary to confirm the validity of this scale for owners speaking different languages and with diverse cultural backgrounds.

This scale paves the way for future studies aiming to develop a common tool for the assessment of both dog– and cat–owner relationships, in terms of cost–benefit balance. Items, such as “How often do you take your pet to visit people?” or “How often do you take your pet in the car?” that had to be excluded from the original C/DORS cat owner scoring process, are no longer present in this new C/DORS version. On the opposite, items that were only included in C/DORS cat scale—that is the CORS—were found to fit the model applied to dog owners, as well.

## Figures and Tables

**Table 1:** Descriptive statistics of the scales used in the study.

Variable	Mean (Min–Max)	Standard Deviation	Skewness	Kurtosis	Shapiro–Wilk Normality Test
**Cat/Dog-Owner Relationship Scale**					
Pet–Owner Interactions	55.68 (24–77)	9.04	−0.21	0.18	0.99 (0.001)
Perceived Emotional Closeness	40.92 (14–49)	6.75	−0.88	0.40	0.92 (<0.001)
Perceived Costs	41.73 (24–49)	5.10	−0.69	0.08	0.95 (<0.001)
**Lexington Attachment to Pets Scale**					
General Attachment	37.58 (23–43)	3.33	−0.93	0.74	0.93 (<0.001)
Animal Rights	18.28 (9–20)	1.90	−1.23	1.36	0.88 (<0.001)
People Substitute	21.61 (9–28)	4.20	−0.44	−0.38	0.96 (<0.001)

**Table 2:** Demographic information of the participants.

Demographic Factor	Mdn (Min–Max)
**Age (years)**	40 (18–76)
**Gender**	**% (N)**
Female	86 (886)
Male	13 (135)
Other/prefer not to say	1 (4)
**Level of education**	
Lower education	5 (47)
Up to secondary degree	44 (456)
Tertiary degree and above	51 (521)
**Occupation**	
Working with animals	25 (251)
Not working with animals/unemployed	63 (637)
Retired	3 (35)
Student	8 (86)
**Geography**	
City (population 200,000+)	45 (458)
Town (population 2000–20,000)	36 (373)
Village (population less than 2000)	8 (86)
Isolated (es. countryside)	10 (105)
**Household composition**	
Single	15 (151)
Couple	42 (429)
3+ people	44 (441)
**Number of previous dogs** (Mdn years, min–max)	2 (0–4)
**Previous experience with dogs**	
Had the first dog at younger than 10 years old	47 (481)
Had the first dog between 10 and 20 years old	22 (225)
Had the first dog at older than 20 years old	31 (319)

**Table 3:** Results of the C/DORS’ EFA and factor loadings. The highest loading for each item is in bold.

	Items	Loadings		
		Pet–Owner Interactions (POI)	Perceived Emotional Closeness (PEC)	Perceived Costs (PC)
4	How often do you kiss your dog?	**0.53**	0.17	0.02
9	How often do you spend time enjoying watching your dog?	**0.58**	−0.07	−0.12
15	How often do you talk to your dog?	**0.65**	0.00	−0.02
21	How often do you cuddle your dog?	**0.79**	0.03	0.02
23	How often do you have your dog with you while relaxing, e.g., watching TV?	**0.51**	0.09	−0.04
26	How often do you pet your dog?	**0.78**	−0.01	0.04
30	How often do you hug your dog?	**0.63**	0.19	0.06
7	How often do you play games with your dog?	**0.49**	−0.06	−0.12
12	How often do you buy your dog presents?	**0.39**	−0.05	0.05
13	How often do you tell your dog things you don’t tell anyone else?	**0.38**	0.22	0.02
28	How often do you give your dog food treats?	**0.41**	−0.17	0.00
31	How often do you groom your dog?	**0.41**	−0.09	−0.06
5	I wish my dog and I never had to be apart.	0.09	**0.53**	0.01
17	I would like to have my dog near me all the time.	0.12	**0.52**	−0.04
18	If everyone else left me, my dog would still be there for me.	−0.04	**0.74**	0.03
20	My dog helps me get through tough times.	0.10	**0.56**	−0.07
22	My dog provides me with constant companionship.	0.01	**0.66**	−0.08
24	My dog is there whenever I need to be comforted.	0.00	**0.80**	0.01
32	My dog is constantly attentive to me.	0.03	**0.66**	−0.03
6	My dog makes too much mess.	−0.01	−0.07	**0.54**
8	It bothers me that my dog stops me doing things I enjoyed before I owned it.	0.01	−0.05	**0.70**
10	It is annoying that sometimes I have to change my plans because of my dog.	−0.02	−0.02	**0.74**
1	How hard is it to look after your dog?	0.06	−0.05	**0.40**
3	There are major aspects of owning a dog I don’t like.	−0.02	−0.05	**0.38**
11	My dog costs too much money.	−0.01	0.04	**0.47**
16	How often does your dog stop you doing things you want to?	0.24	−0.14	**0.34**
2	My dog gives me a reason to get up in the morning	-	-	-
14	How often do you feel that looking after your dog is a chore?	-	-	-
19	How often do you feel that having a dog is more trouble than it’s worth?	-	-	-
25	How traumatic do you think it will be for you when your dog dies?	-	-	-
27	How often do you take your dog to visit people?	-	-	-
29	How often do you take your dog in the car?	-	-	-

**Table 4:** Test–retest reliability (*n* = 31).

Item	T_0_ Median (Range)	T_1_ Median (Range)	Spearman Rho	*p*
**Lexington Attachment to Pets Scale**				
General Attachment	36.5 (26–41)	36.5 (26–41)	0.78	<0.001
People Substitute	18 (11–27)	19 (11–27)	0.89	<0.001
Animal Rights	17 (12–20)	17 (13–20)	0.78	<0.001
**Cat/Dog-Owner Relationship Scale**				
Pet–Owner Interactions	51 (33–67)	51 (31–65)	0.85	<0.001
Perceived EmotionalCloseness	37 (28–49)	36 (26–47)	0.82	<0.001
Perceived Costs	15 (7–29)	15 (7–24)	0.67	<0.001

**Table 5:** Spearman’s correlations between LAPS and C/DORS (Spearman rho, *p* values).

	Pet–Owner Interactions	Perceived Emotional Closeness	Perceived Costs
General Attachment	0.45, <0.001	0.58, <0.001	0.26, <0.001
People Substitute	0.45, <0.001	0.67, <0.001	0.22, <0.001
Animal Rights	0.36, <0.001	0.46, <0.001	0.23, <0.001

**Table 6:** Variables selections for the C/DORS regression model.

Predictors	Pet–Owner Interactions	Perceived Emotional Closeness	Perceived Costs
Owner’s age ^a^	r = −0.09, *p* = 0.005	**r = −0.15, *p* < 0.001**	r = 0.03, *p* = 0.410
Owner’s gender ^b^	χ^2^ (df = 2) = 9.86, *p* = 0.007	**χ^2^ (df = 2) = 18.24, *p* < 0.001**	χ^2^ (df = 2) = 5.73, *p* = 0.057
Level of education ^b^	χ^2^ (df = 3) = 3.03, *p* = 0.388	χ^2^ (df = 3) = 4.36, *p* = 0.225	χ^2^ (df = 3) = 5.47, *p* = 0.140
Occupation ^b^	**χ^2^ (df = 4) = 25.69, *p* < 0.001**	**χ^2^ (df = 4) = 37.48, *p* < 0.001**	χ^2^ (df = 4) = 8.89, *p* = 0.064
Geography ^b^	χ^2^ (df = 4) = 0.97, *p* = 0.913	χ^2^ (df = 4) = 3.20, *p* = 0.525	χ^2^ (df = 4) = 5.83, *p* = 0.212
Household composition ^b^	χ^2^ (df = 5) = 5.66, *p* = 0.341	χ^2^ (df = 5) = 15.71, *p* = 0.008	χ^2^ (df = 5) = 4.413, *p* = 0.492
Number of previous dogs ^a^	r = −0.03, *p* = 0.329	r = −0.03, *p* = 0.288	r = 0.09, *p* = 0.004
Previous experience with dogs ^b^	χ^2^ (df = 2) = 4.73, *p* = 0.094	χ^2^ (df = 2) = 5.42, *p* = 0.066	**χ^2^ (df = 2) = 13.85, *p* < 0.001**
Dog’s age ^a^	**r = −0.12, *p* < 0.001**	r = 0.07, *p* = 0.045	**r = 0.12, *p* < 0.001**
Dog’s age at adoption ^a^	r = −0.07, *p* = 0.047	r = −0.08, *p* = 0.015	r = 0.02, *p* = 0.628
Dog’s sex ^c^	W = 104,298, *p* = 0.423	W = 107,578, *p* = 0.100	**W = 114,507, *p* < 0.001**
Neutering status ^c^	W = 102,202, *p* = 0.253	W = 104,768, *p* = 0.069	W = 88,950, *p* = 0.020
Age when neutered ^b^	χ^2^ (df = 4) = 5.14, *p* = 0.273	χ^2^ (df = 4) = 3.74, *p* = 0.442	χ^2^ (df = 4) = 12.39, *p* = 0.015
Weight ^a^	r = −0.04, *p* = 0.190	r = −0.07, *p* = 0.047	r = −0.09, *p* = 0.006
Breeds ^b^	χ^2^ (df = 87) = 84.36, *p* = 0.560	χ^2^ (df = 87) = 98.92, *p* = 0.180	χ^2^ (df = 87) = 101.66, *p* = 0.135
Origin of the dog ^b^	χ^2^ (df = 7) = 8.54, *p* = 0.287	χ^2^ (df = 7) = 10.20, *p* = 0.177	χ^2^ (df = 7) = 9.04, *p* = 0.249
Activities with the dog ^b^	χ^2^ (df = 4) = 9.47, *p* = 0.050	χ^2^ (df = 4) = 11.06, *p* = 0.026	χ^2^ (df = 4) = 9.53, *p* = 0.049
Health issues ^c^	W = 77,720, *p* = 0.719	W = 77,131, *p* = 0.852	W = 79,932, *p* = 0.310
Behaviour issues ^c^	W = 64,532, *p* = 0.549	**W = 71,020, *p* < 0.001**	**W = 74,492, *p* < 0.001**
Living space ^b^	**χ^2^ (df = 2) = 36.86, *p* < 0.001**	χ^2^ (df = 2) = 3.76, *p* = 0.153	χ^2^ (df = 2) = 6.54, *p* = 0.038
Other pets ^b^	χ^2^ (df = 3) = 3.99, *p* = 0.262	χ^2^ (df = 3) = 4.46, *p* = 0.216	χ^2^ (df = 3) = 7.48, *p* = 0.058

Note: bold = *p* < 0.001 (corrected alpha level of 0.05 over 42 comparisons), ^a^ = Spearman correlation, ^b^ = Kruskall–Wallis test, ^c^ *=* Mann–Whitney U test.

**Table 7:** Ordinal regressions for the response variables C/DORS Pet–Owner Interactions, Perceived Emotional Closeness and Perceived Costs.

Dependent Variable	Parameter	B	Std. Error	Sig.	Exp(B)	95% Wald Confidence Interval for Exp(B)	
						Lower	Upper
Pet–Owner Interactions	[Occupation = working with animals]	−0.931	0.2200	<0.0001	0.394	0.256	0.607
	[Occupation = retired]	−0.952	0.3667	0.009	0.386	0.188	0.792
	[Occupation = student]	0 ^a^				1	
	[Living space = indoors and outdoors]	1.226	0.3848	0.001	3.408	1.603	7.246
	[Living space = indoors]	1.417	0.2021	0.000	4.126	2.776	6.131
	[Living space = outdoors]	0 ^a^			1		
Perceived Emotional Closeness	[Occupation = working with animals]	−1.172	0.2293	0.000	0.310	0.198	0.485
	[Occupation = not working with animals /unemployed]	−0.448	0.2066	0.030	0.639	0.426	0.958
	[Occupation = retired]	−1.053	0.3483	0.003	0.349	0.176	0.691
	[Occupation = student]	0 ^a^			1		
	[Activities = AAI/assistance]	−1.446	0.5494	0.009	0.236	0.080	0.692
	[Activities = none]	0 ^a^			1		
	[Behaviour problems = no]	0.434	0.1470	0.003	1.543	1.157	2.058
	[Behaviour problems = yes]	0 ^a^			1		
Perceived costs	[Behaviour problems = no]	0.470	0.1439	0.001	1.600	1.207	2.122
	[Behaviour problems = yes]	0 ^a^			1		

Significance: *p* < 0.05 (only parameters with *p* < 0.05 are reported). B: regression coefficient. SE: standard error of the mean, OR: odds ratio, CI: confidence interval. ^a^ This parameter was set to zero because it is the term of comparison.

## Data Availability

The data presented in this study are available on request from the corresponding author.

## Supplementary Materials

The following are available online at https://www.mdpi.com/article/10.3390/ani11082166/s1, Table S1: C/DORS original English version (already adapted to dogs) and the Italian translation used in the current study, Table S2: LAPS original English version (already adapted to dogs) and the Italian translation used in the current study, Table S3: Original response 1–5 scale, 1–7 scale English adaptation and final 1–7 scale Italian translation, Table S4: Demographic information of the dogs.
