# Supplementary material for: The Dog–Owner Relationship: Refinement and Validation of the Italian C/DORS for Dog Owners and Correlation with the LAPS
Source: Animals (Basel). 2021 Jul 22;11(8):2166. doi: 10.3390/ani11082166 (PMC8388506; doi:10.3390/ani11082166)
Supplement: Supplementary file 1 [file animals-11-02166-s001.zip › animals-1297451-supplementary.pdf]

Table S1. C/DORS original English version (already adapted to dogs) and the Italian translation used in the current study.

Item n.	Original English version	Italian translation
1	How hard is it to look after your dog?	Quanto è difficile prenderti cura del tuo cane?
2	My dog gives me a reason to get up in the morning.	Il mio cane mi dà un buon motivo per alzarmi al mattino
3	There are major aspects of owning a dog I don't like.	Ci sono molti aspetti del possedere un cane che non mi piacciono
4	How often do you kiss your dog?	Con che frequenza baci il tuo cane?
5	I wish my dog and I never had to be apart.	Vorrei che il mio che ed io non dovessimo mai stare lontani
6	My dog makes too much mess.	Il mio cane crea troppo disordine/sporcizia
7	How often do you play games with your dog?	Con che frequenza giochi con il tuo cane?
8	It bothers me that my dog stops me doing things I enjoyed before I owned it.	Mi secca che il mio cane mi impedisca di fare cose che mi piaceva fare prima che lo avessi
9	How often do you spend time enjoying watching your dog?	Con che frequenza ti diverti ad osservare il tuo cane?
10	It is annoying that sometimes I have to change my plans because of my dog.	È seccante che a volte debba cambiare i miei piani a causa del mio cane
11	My dog costs too much money.	Il mio cane mi costa troppi soldi
12	How often do you buy your dog presents?	Con che frequenza compri regali per il tuo cane?
13	How often do you tell your dog things you don't tell anyone else?	Con che frequenza dici al tuo cane cose che non dici a nessun altro?
14	How often do you feel that looking after your dog is a chore?	Quanto spesso hai la sensazione che prenderti cura del tuo cane sia un'incombenza?
15	How often do you talk to your dog?	Con che frequenza parli con il tuo cane?
16	How often does your dog stop you doing things you want to?	Quanto spesso il tuo cane ti impedisce di fare cose che vorresti?
17	I would like to have my dog near me all the time.	Vorrei avere il mio cane sempre vicino a me
18	If everyone else left me, my dog would still be there for me.	Se tutti mi lasciassero, il mio cane sarebbe comunque lì per me
19	How often do you feel that having a dog is more trouble than it's worth?	Quanto frequentemente hai la sensazione che avere un cane sia più un problema che qualcosa che valga la pena?
20	My dog helps me get through tough times.	Il mio cane mi aiuta a superare i momenti difficili
21	How often do you cuddle your dog?	Con che frequenza coccoli il tuo cane?
22	My dog provides me with constant companionship.	Il mio cane mi fa sempre compagnia
23	How often do you have your dog with you while relaxing, e.g., watching TV?	Quanto spesso hai il tuo cane con te mentre ti rilassi (es. guardando la tv)?
24	My dog is there whenever I need to be comforted.	Il mio cane c'è ogni volta che ho bisogno di essere confortato
25	How traumatic do you think it will be for you when your dog dies?	Quanto pensi sarà traumatica per te la morte del tuo cane?
26	How often do you pet your dog?	Quanto spesso accarezzi il tuo cane?
27	How often do you take your dog to visit people?	Con che frequenza porti il tuo cane a far visita ad altre persone?

28	How often do you give your dog food treats?	Con che frequenza dai bocconcini appetitosi al tuo cane?
29	How often do you take your dog in the car?	Con che frequenza porti il tuo cane in auto?
30	How often do you hug your dog?	Con che frequenza abbracci il tuo cane?
31	How often do you groom your dog?	Con che frequenza ti prendi cura del pelo del tuo cane?
32	My dog is constantly attentive to me.	Il mio cane è molto premuroso con me

Table S2. LAPS original English version (already adapted to dogs) and the Italian translation used in the current study.

Item n.	Original English version	Italian translation
1	My dog means more to me than any of my friends	Il mio cane significa molto per me, più di qualsiasi amico/a
2	Quite often I confide in my dog	Molto spesso confido nel mio cane
3	I believe that dogs should have the same rights and privileges as family members	Credo che i cani dovrebbero avere gli stessi diritti e privilegi degli altri membri della famiglia
4	I believe my dog is my best friend	Credo che il mio cane sia il mio migliore amico
5	Quite often, my feelings towards people are affected by how they react to my dog	Molto spesso i miei sentimenti verso le persone sono influenzati dal modo in cui reagiscono al mio cane
6	I love my dog because he/she is more loyal to me than most of the people in my life	Amo il mio cane perché mi è più fedele della maggior parte delle persone della mia vita
7	I enjoy showing other people pictures of my dog	Mi piace mostrare agli altri le fotografie del mio cane
8	I think my dog is just a dog	Penso che il mio cane sia solo un animale
9	I love my dog because it never judges me	Amo il mio cane perché non mi giudica mai
10	My dog knows when I'm feeling bad	Il mio cane sa quando sto male
11	I often talk to other people about my dog	Spesso parlo agli altri del mio cane
12	My dog understands me	Il mio cane mi capisce
13	I believe that loving my dog helps me stay healthy	Credo che amare il mio cane mi aiuti a rimanere in salute
14	Dogs deserve as much respect as humans do	Gli animali domestici meritano rispetto tanto quanto gli esseri umani
15	My dog and I have a very close relationship	Io e il mio cane abbiamo un rapporto molto stretto
16	I would do almost anything to take care of my dog	Farei quasi qualsiasi cosa per prendermi cura del mio cane
17	I play with my dog quite often	Gioco abbastanza spesso con il mio cane
18	I consider my dog to be a great companion	Considero il mio cane un ottimo compagno
19	My dog makes me feel happy	Il mio cane mi rende felice
20	I feel that my dog is a part of my family	Ritengo che il mio cane faccia parte della mia famiglia
21	I am not very attached to my dog	Non sono molto attaccato/a al mio cane
22	Owning a dog adds to my happiness	Avere un cane accresce la mia felicità
23	I consider my dog to be a friend	Considero il mio cane un amico

Table S3. Original response 1 to 5 scale, 1 to 7 scale English adaptation and final 1 to 7 scale Italian translation. Items that had to be reverse scored in the Italian version are marked with (R).

Item n.	Original English 1 to 5 scale	English 1 to 7 scale	Italian 1 to 7 scale
1 (R)	Very hard/Hard/Neither hard nor easy/Easy/Very easy	Very easy/Easy/ Somewhat easy/Neither hard nor easy/Somewhat hard/Hard/Very hard	Molto facile/Facile/Abbastanza facile/Né difficile né facile/Abbastanza difficile/Difficile/Molto difficile
2	Strongly agree/Agree/Neither agree nor disagree/Disagree/Strongly disagree	Strongly disagree/Disagree/Somewhat disagree/Neither agree nor disagree/Somewhat agree/Agree/Strongly agree	Completamente in disaccordo/In disaccordo/ Abbastanza in disaccordo/Né d'accordo né in disaccordo/Abbastanza d'accordo/D'accordo/Completamente d'accordo
3 (R)	Strongly agree/Agree/Neither agree nor disagree/Disagree/Strongly disagree	Strongly disagree/Disagree/Somewhat disagree/Neither agree nor disagree/Somewhat agree/Agree/Strongly agree	Completamente in disaccordo/In disaccordo/ Abbastanza in disaccordo/Né d'accordo né in disaccordo/Abbastanza d'accordo/D'accordo/Completamente d'accordo
4	At least once a day/Once every few days/Once a week/Once a month/ Never	Once a month or less/About once a week/A few times a week/About once a day/More than once a day/About once an hour/More than once an hour	Una volta al mese o meno/Circa una volta a settimana/Qualche volta a settimana/Circa una volta al giorno/Più volte al giorno/ circa una volta all'ora/Più volte all'ora
5	Strongly agree/Agree/Neither agree nor disagree/Disagree/Strongly disagree	Strongly disagree/Disagree/Somewhat disagree/Neither agree nor disagree/Somewhat agree/Agree/Strongly agree	Completamente in disaccordo/In disaccordo/ Abbastanza in disaccordo/Né d'accordo né in disaccordo/Abbastanza d'accordo/D'accordo/Completamente d'accordo
6 (R)	Strongly agree/Agree/Neither agree nor disagree/Disagree/Strongly disagree	Strongly disagree/Disagree/Somewhat disagree/Neither agree nor disagree/Somewhat agree/Agree/Strongly agree	Completamente in disaccordo/In disaccordo/ Abbastanza in disaccordo/Né d'accordo né in disaccordo/Abbastanza d'accordo/D'accordo/Completamente d'accordo
7	At least once a day/Once every few days/Once a week/Once a month/ Never	Once a month or less/About once a week/A few times a week/About once a day/More than once a day/About once an hour/More than once an hour	Una volta al mese o meno/Circa una volta a settimana/Qualche volta a settimana/Circa una volta al giorno/Più volte al giorno/ circa una volta all'ora/Più volte all'ora
8 (R)	Strongly agree/Agree/Neither agree nor disagree/Disagree/Strongly disagree	Strongly disagree/Disagree/Somewhat disagree/Neither agree nor disagree/Somewhat agree/Agree/Strongly agree	Completamente in disaccordo/In disaccordo/ Abbastanza in disaccordo/Né d'accordo né in disaccordo/Abbastanza d'accordo/D'accordo/Completamente d'accordo
9	At least once a day/Once a week/ Once	Once a month or less/About once a week/A few times a week/About once a day/More than once a	Una volta al mese o meno/Circa una volta a settimana/Qualche volta a settimana/Circa una volta al

	a month/ A couple of times a year/Never	day/About once an hour/More than once an hour	giorno/Più volte al giorno/ circa una volta all'ora/Più volte all'ora
10 (R)	Strongly agree/Agree/Neither agree nor disagree/Disagree/Strongly disagree	Strongly disagree/Disagree/Somewhat disagree/Neither agree nor disagree/Somewhat agree/Agree/Strongly agree	Completamente in disaccordo/In disaccordo/ Abbastanza in disaccordo/Né d'accordo né in disaccordo/Abbastanza d'accordo/D'accordo/Completamente d'accordo
11 (R)	Strongly agree/Agree/Neither agree nor disagree/Disagree/Strongly disagree	Strongly disagree/Disagree/Somewhat disagree/Neither agree nor disagree/Somewhat agree/Agree/Strongly agree	Completamente in disaccordo/In disaccordo/ Abbastanza in disaccordo/Né d'accordo né in disaccordo/Abbastanza d'accordo/D'accordo/Completamente d'accordo
12	At least once a day/Once a week/ Once a month/ A couple of times a year/Never	Once a year or less/A few times a year/About once a month/About once a week/A few times a week/About once a day/More than once a day	Una volta all'anno o meno/Qualche volta all'anno/Circa una volta al mese/Circa una volta a settimana/Qualche volta alla settimana/ circa una volta al giorno/Più volte al giorno
13	Once a day/Once a week/Once a month/Once a year/Never	Once a year or less/A few times a year/About once a month/About once a week/A few times a week/About once a day/More than once a day	Una volta all'anno o meno/Qualche volta all'anno/Circa una volta al mese/Circa una volta a settimana/Qualche volta alla settimana/ circa una volta al giorno/Più volte al giorno
14 (R)	Once a day/Once a week/Once a month/Once a year/Never	Once a month or less/About once a week/A few times a week/About once a day/More than once a day/About once an hour/More than once an hour	Una volta al mese o meno/Circa una volta a settimana/Qualche volta a settimana/Circa una volta al giorno/Più volte al giorno/ circa una volta all'ora/Più volte all'ora
15	At least once a day/Once every few days/Once a week/Once a month/ Never	Once a month or less/About once a week/A few times a week/About once a day/More than once a day/About once an hour/More than once an hour	Una volta al mese o meno/Circa una volta a settimana/Qualche volta a settimana/Circa una volta al giorno/Più volte al giorno/ circa una volta all'ora/Più volte all'ora
16 (R)	Once a day/Once a week/Once a month/Once a year/Never	Once a month or less/About once a week/A few times a week/About once a day/More than once a day/About once an hour/More than once an hour	Una volta al mese o meno/Circa una volta a settimana/Qualche volta a settimana/Circa una volta al giorno/Più volte al giorno/ circa una volta all'ora/Più volte all'ora
17	Strongly agree/Agree/Neither agree nor disagree/Disagree/Strongly disagree	Strongly disagree/Disagree/Somewhat disagree/Neither agree nor disagree/Somewhat agree/Agree/Strongly agree	Completamente in disaccordo/In disaccordo/ Abbastanza in disaccordo/Né d'accordo né in disaccordo/Abbastanza d'accordo/D'accordo/Completamente d'accordo
18	Strongly agree/Agree/Neither agree nor	Strongly disagree/Disagree/Somewhat disagree/Neither agree nor	Completamente in disaccordo/In disaccordo/ Abbastanza in disaccordo/Né d'accordo né in disaccordo/Abbastanza

	disagree/Disagree/Strongly disagree	disagree/Somewhat agree/Agree/Strongly agree	d'accordo/D'accordo/Completamente d'accordo
19 (R)	Once a day/Once a week/Once a month/Once a year/Never	Once a month or less/About once a week/A few times a week/About once a day/More than once a day/About once an hour/More than once an hour	Una volta al mese o meno/Circa una volta a settimana/Qualche volta a settimana/Circa una volta al giorno/Più volte al giorno/ circa una volta all'ora/Più volte all'ora
20	Strongly agree/Agree/Neither agree nor disagree/Disagree/Strongly disagree	Strongly disagree/Disagree/Somewhat disagree/Neither agree nor disagree/Somewhat agree/Agree/Strongly agree	Completamente in disaccordo/In disaccordo/ Abbastanza in disaccordo/Né d'accordo né in disaccordo/Abbastanza d'accordo/D'accordo/Completamente d'accordo
21	At least once a day/Once every few days/Once a week/Once a month/ Never	Once a month or less/About once a week/A few times a week/About once a day/More than once a day/About once an hour/More than once an hour	Una volta al mese o meno/ Circa una volta a settimana/ Qualche volta a settimana/ Circa una volta al giorno/ Più volte al giorno/ Circa una volta all'ora/ Più volte all'ora
22	Strongly agree/Agree/Neither agree nor disagree/Disagree/Strongly disagree	Strongly disagree/Disagree/Somewhat disagree/Neither agree nor disagree/Somewhat agree/Agree/Strongly agree	Completamente in disaccordo/In disaccordo/ Abbastanza in disaccordo/Né d'accordo né in disaccordo/Abbastanza d'accordo/D'accordo/Completamente d'accordo
23	At least once a day/Once every few days/Once a week/Once a month/ Never	Once a month or less/About once a week/A few times a week/About once a day/More than once a day/About once an hour/More than once an hour	Una volta al mese o meno/ Circa una volta a settimana/ Qualche volta a settimana/ Circa una volta al giorno/ Più volte al giorno/ Circa una volta all'ora/ Più volte all'ora
24	Strongly agree/Agree/Neither agree nor disagree/Disagree/Strongly disagree	Strongly disagree/Disagree/Somewhat disagree/Neither agree nor disagree/Somewhat agree/Agree/Strongly agree	Completamente in disaccordo/In disaccordo/ Abbastanza in disaccordo/Né d'accordo né in disaccordo/Abbastanza d'accordo/D'accordo/Completamente d'accordo
25	Very traumatic/Traumatic/Neither traumatic nor untraumatic/Untraumatic/Very untraumatic	A big relief/a relief/ a little relief/ Neither traumatic nor a relief/a little traumatic/ traumatic/very traumatic	Un grande sollievo/ Un sollievo/ Un piccolo sollievo/Né un sollievo né un trauma/Un po' traumatico/Traumatico/ Molto traumatico
26	At least once a day/Once every few days/Once a week/Once a month/ Never	Once a month or less/About once a week/A few times a week/About once a day/More than once a day/About once an hour/More than once an hour	Una volta al mese o meno/Circa una volta a settimana/Qualche volta a settimana/Circa una volta al giorno/Più volte al giorno/ circa una volta all'ora/Più volte all'ora
27	At least once a day/Once a week/ Once a month/ A couple of times a year/Never	Once a year or less/A few times a year/About once a month/About once a week/A few times a week/About once a day/More than once a day	Una volta all'anno o meno/Qualche volta all'anno/Circa una volta al mese/Circa una volta a settimana/Qualche volta alla settimana/ circa una volta al giorno/Più volte al giorno

28	At least once a day/Once every few days/Once a week/Once a month/ Never	Once a month or less/About once a week/A few times a week/About once a day/More than once a day/About once an hour/More than once an hour	Una volta al mese o meno/ circa una volta a settimana/ qualche volta a settimana/ circa una volta al giorno/ più volte al giorno/ circa una volta all'ora/ più volte all'ora
29	At least once a day/Once every few days/Once a week/Once a month/ Never	Once a year or less/A few times a year/About once a month/About once a week/A few times a week/About once a day/More than once a day	Una volta al mese o meno/Circa una volta a settimana/Qualche volta a settimana/Circa una volta al giorno/Più volte al giorno/ circa una volta all'ora/Più volte all'ora
30	At least once a day/Once every few days/Once a week/Once a month/ Never	Once a month or less/About once a week/A few times a week/About once a day/More than once a day/About once an hour/More than once an hour	Una volta al mese o meno/Circa una volta a settimana/Qualche volta a settimana/Circa una volta al giorno/Più volte al giorno/ circa una volta all'ora/Più volte all'ora
31	At least once a day/Once every few days/Once a week/Once a month/ Never	Once a year or less/A few times a year/About once a month/About once a week/A few times a week/About once a day/More than once a day	Una volta all'anno o meno/Qualche volta all'anno/Circa una volta al mese/Circa una volta a settimana/Qualche volta alla settimana/ circa una volta al giorno/Più volte al giorno
32	Strongly agree/Agree/Neither agree nor disagree/Disagree/Strongly disagree	Strongly disagree/Disagree/Somewhat disagree/Neither agree nor disagree/Somewhat agree/Agree/Strongly agree	Completamente in disaccordo/In disaccordo/ Abbastanza in disaccordo/Né d'accordo né in disaccordo/Abbastanza d'accordo/D'accordo/Completamente d'accordo

Table S4. Demographic information of the dogs

**Demographic factor**	
	**Mdn (min – max)**
**Age** (years)	7 (<0.5-20)
**Age at adoption** (years)	1 (0-16)
**Weight** (Kg)	19 (1-90)
**Sex**	**% (N)**
Female	53 (540)
Male	47 (486)
**Neutering status**	
Neutered	59 (604)
Intact	41 (422)
**Age when neutered**	

Before 3 years	42 (429)
After 3 years	16 (164)
Never	42 (422)
**Breeds**	
American Staffordshire Terrier	1 (9)
Australian Shepherd	1 (11)
Beagle	<1 (6)
Bernese Mountain Dog	1 (11)
Bloodhound	<1 (7)
Border Collie	4 (37)
Boxer	<1 (7)
Cavalier King Charles Spaniel	1 (9)
Cocker Spaniel	1 (11)
Dachshund	2 (19)
French Bulldog	<1 (7)
German Shepherd	2 (21)
Golden Retriever	2 (20)
Irish setter	1 (11)
Jack Russell Terrier	3 (30)
Labrador Retriever	5 (46)
Maltese	<1 (7)
Malinois	<1 (7)
Maremmano-Abruzzese Sheepdog	<1 (6)
Miniature Pinscher	1 (11)
Pitbull	1 (9)
Poodle	<1 (7)
Scottish Shepherd	<1 (7)
Siberian Husky	<1 (7)
Other Breeds[a]	15 (154)

Mixed Breed	54 (550)
**Origin of the dog**	
Professional breeder	16 (161)
Private (e.g., friends, acquaintances, family breeder)	40 (410)
Given as a gift	3 (31)
Born at home	2 (24)
Pet shop	0 (4)
Found / Shelter / Sanctuary	38 (383)
**Activities with the dog**	
None (my dog is only a companion)	68 (696)
Training (at home, without competing)	36 (222)
Sport (competing)	7 (70)
Work (hunting, herding, search & rescue, etc.)	3 (27)
Animal Assisted Interventions	1 (11)
**Health issues**	
Yes	25 (253)
No	75 (773)
**Behaviour issues**	
Yes	18 (187)
No	82 (84)
**Living space**	
Indoor	89 (913)
Outdoor only	8 (86)
Indoor and outdoor	3 (27)
**Other pets**	
None	36 (365)
Only dogs	28 (287)
Only cats	20 (203)
Dogs and cats	27 (171)

[a] Breeds with frequency below 5 have been add together.
